# Supplementary material for: A bright idea—metabarcoding arthropods from light fixtures
Source: PeerJ. 2021 Jul 26;9:e11841. doi: 10.7717/peerj.11841 (PMC8320520; doi:10.7717/peerj.11841)
Supplement: Supplemental Information 1 [file peerj-09-11841-s001.pdf]

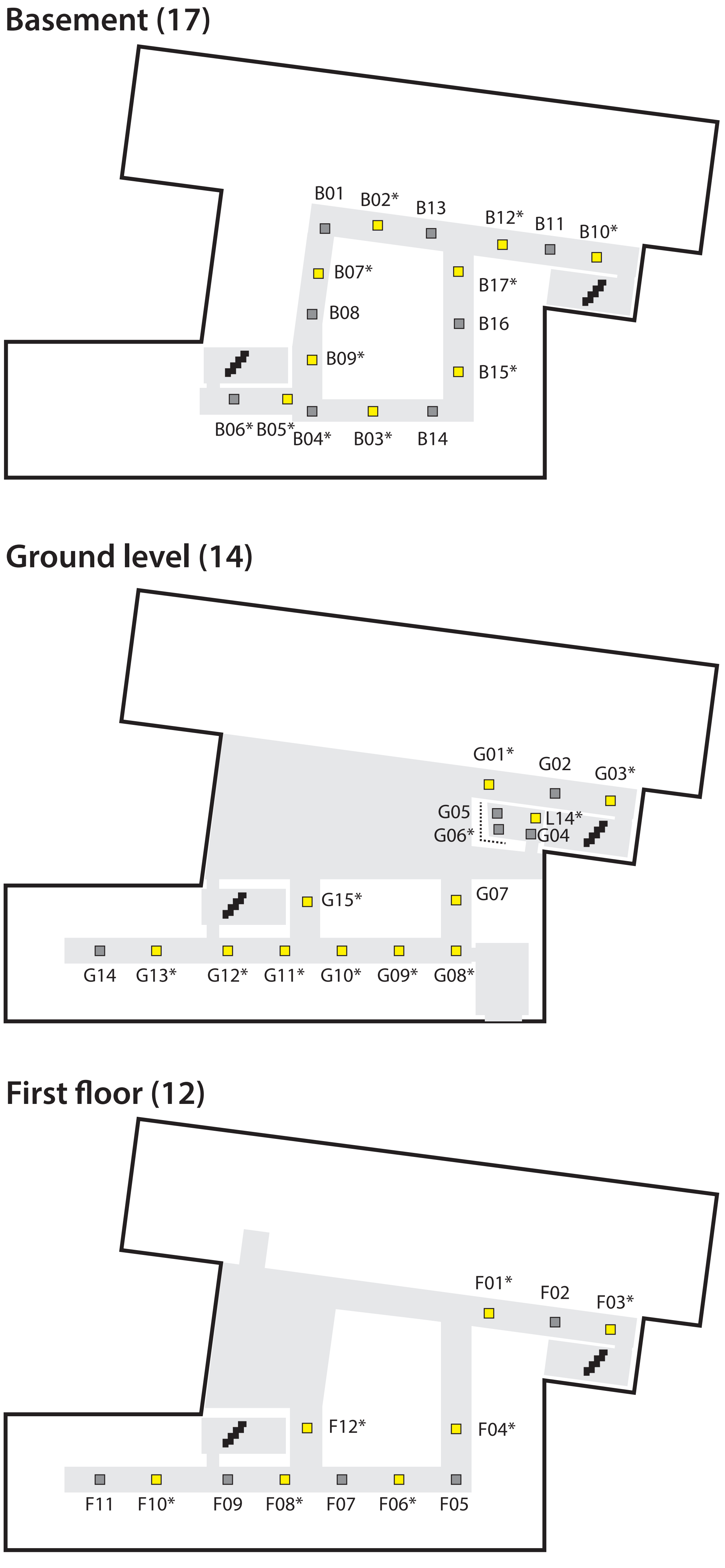

Fig S1: Floor plans of the CBG, with sampled light fixtures indicated by squares. Light fixtures that are turned off at night are indicated in grey, with light fixtures that remain on over night indicated in yellow. Sample IDs with an asterisk (*) indicate samples that had a large amount of biomass and where ground in 20 ml tubes, while samples without an asterisk where ground in 2 ml tubes.
