# Supplementary figures and images for: A bright idea—metabarcoding arthropods from light fixtures
Source: PeerJ. 2021 Jul 26;9:e11841. doi: 10.7717/peerj.11841 (PMC8320520; doi:10.7717/peerj.11841)

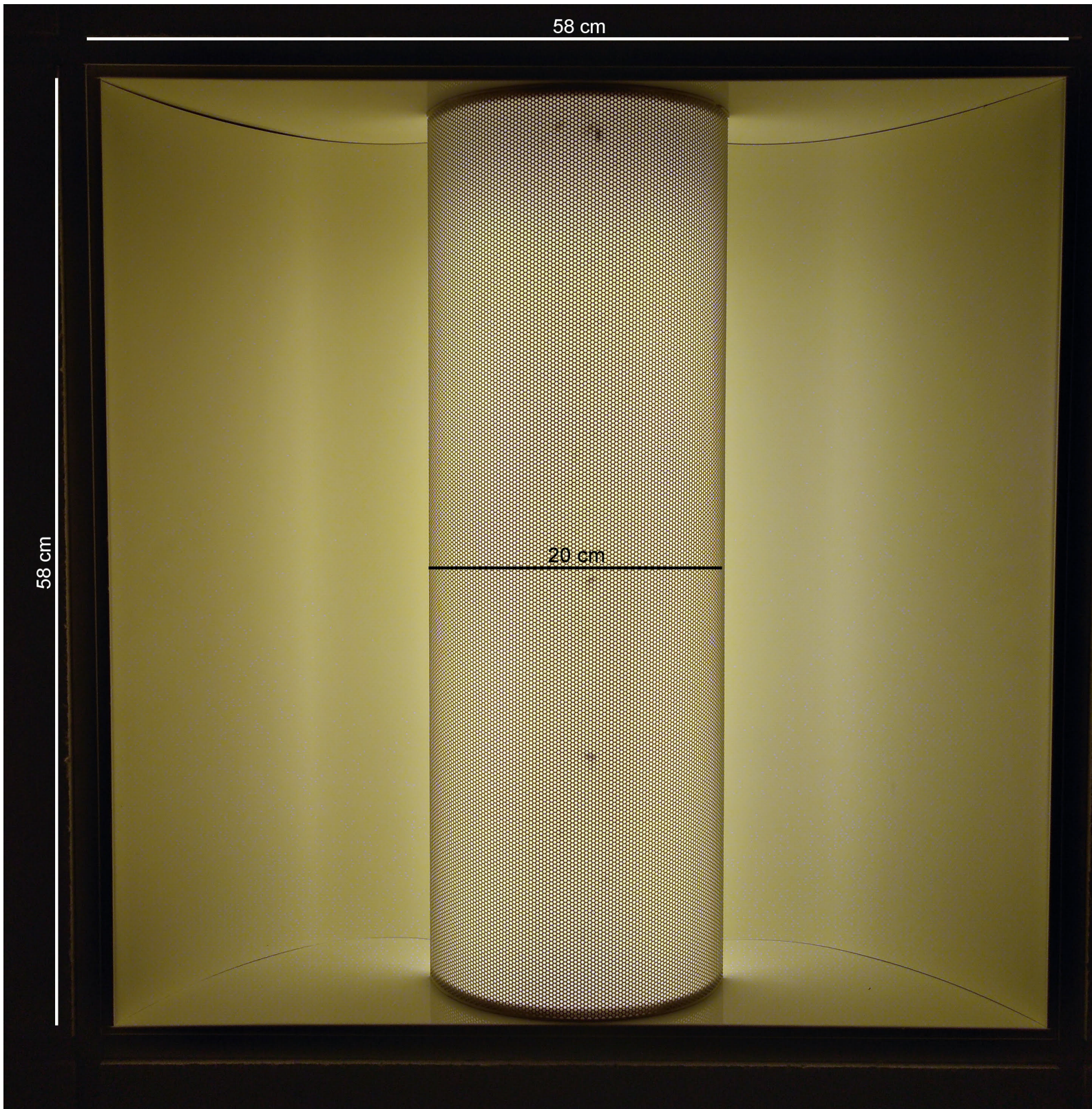

**Fig S1:** Picture of the light fixtures at the CBG.

Supplement: Supplemental Information 2 [file peerj-09-11841-s002.pdf]
