# Supplementary material for: A bright idea—metabarcoding arthropods from light fixtures
Source: PeerJ. 2021 Jul 26;9:e11841. doi: 10.7717/peerj.11841 (PMC8320520; doi:10.7717/peerj.11841)
Supplement: Supplemental Information 3 [file peerj-09-11841-s003.pdf]

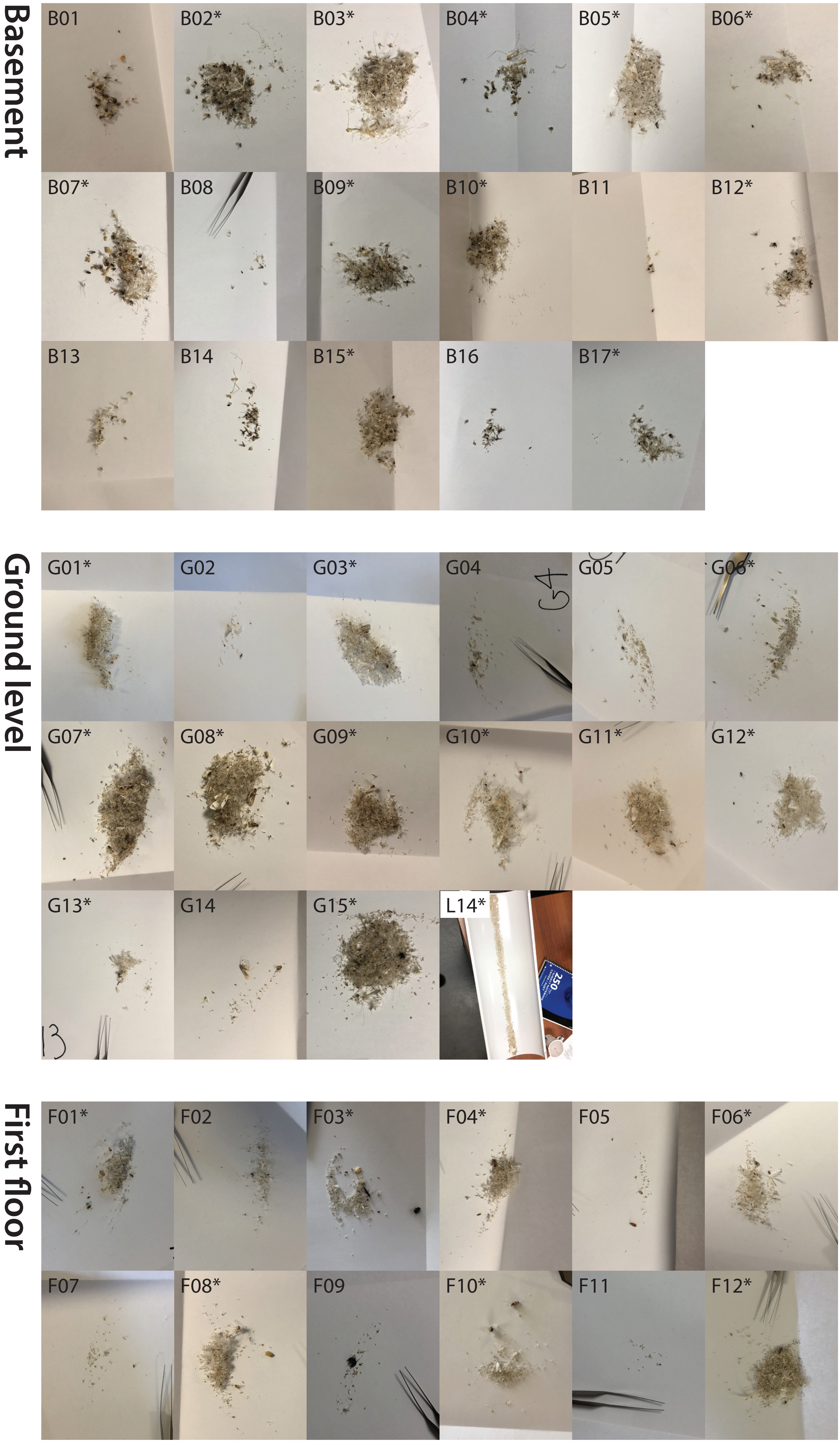

Fig S3: Pictures of arthropods collected at the CBG. Sample IDs marked with asterisks (*) are ground in 20 ml tubes, ones without in 2 ml tubes.
