# Supplementary material for: A bright idea—metabarcoding arthropods from light fixtures
Source: PeerJ. 2021 Jul 26;9:e11841. doi: 10.7717/peerj.11841 (PMC8320520; doi:10.7717/peerj.11841)
Supplement: Supplemental Information 5 [file peerj-09-11841-s005.pdf]

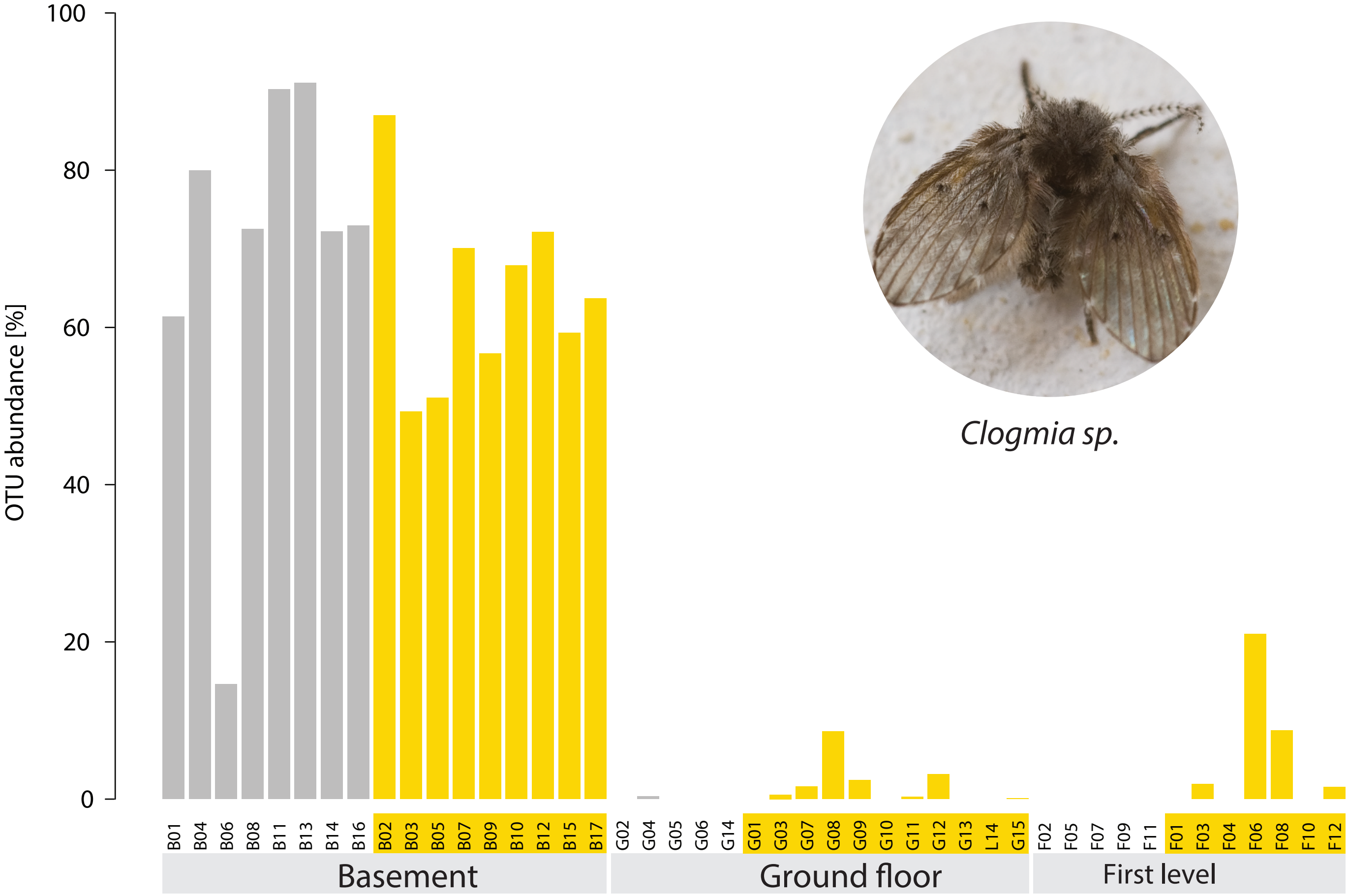

Fig S5: Barplot showing proportions of Clogmia sp. across all floors and samples collected at CBG. Samples where the light fixtures were turned on overnight are coloured yellow, samples from fixtures only switched on during the day are coloured grey. Picture: Mvuijlst, CC BY-SA 3.0.
