# Supplementary material for: A bright idea—metabarcoding arthropods from light fixtures
Source: PeerJ. 2021 Jul 26;9:e11841. doi: 10.7717/peerj.11841 (PMC8320520; doi:10.7717/peerj.11841)
Supplement: Supplemental Information 6 [file peerj-09-11841-s006.pdf]

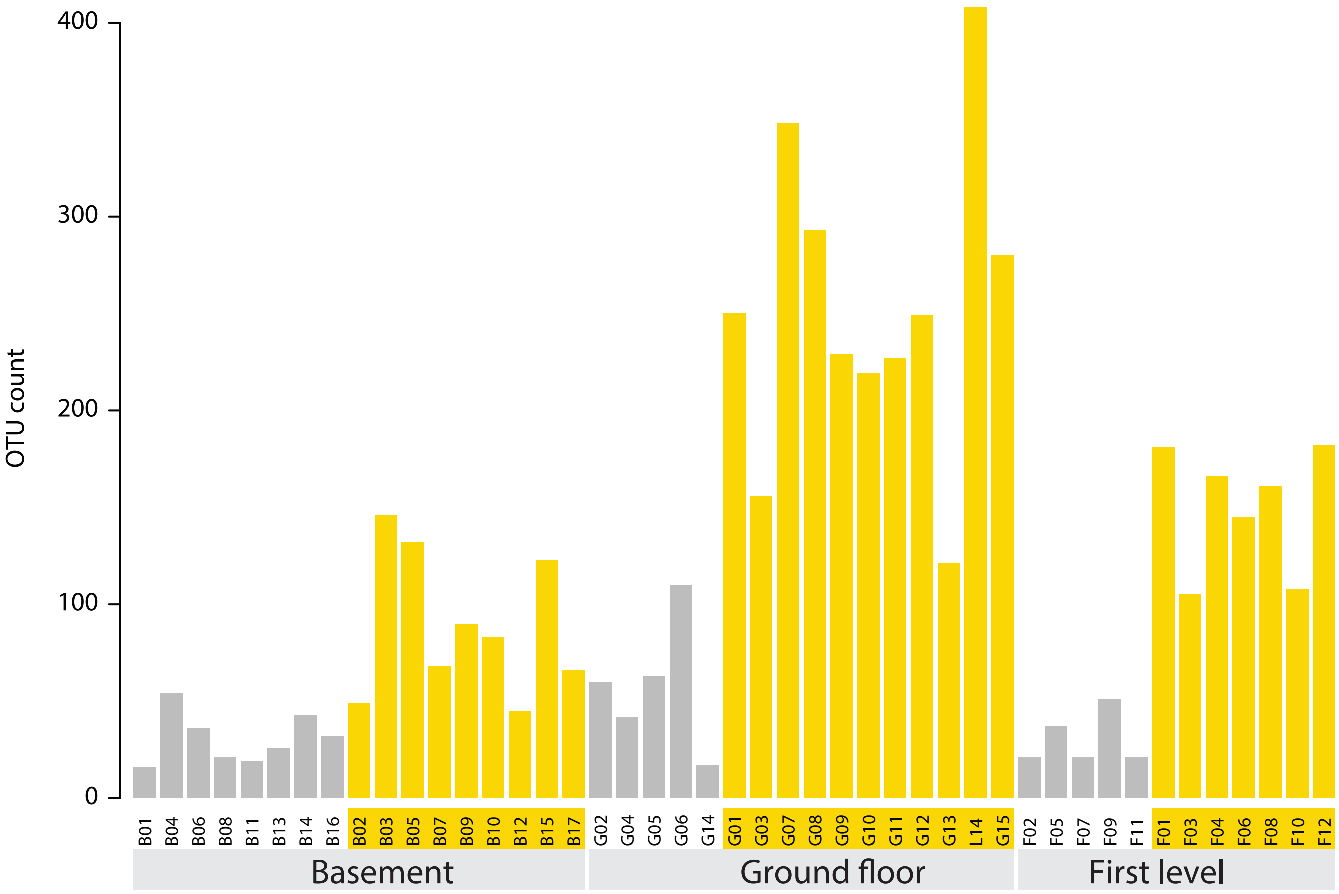

Fig S6: OTU counts in each sample collected at CBG. Samples highlighted in yellow indicate light fixtures that stay turned on overnight, while bars in gray indicate OTU abundance for light fixtures that are turned off overnight.
