# Supplementary material for: A bright idea—metabarcoding arthropods from light fixtures
Source: PeerJ. 2021 Jul 26;9:e11841. doi: 10.7717/peerj.11841 (PMC8320520; doi:10.7717/peerj.11841)
Supplement: Supplemental Information 4 [file peerj-09-11841-s004.pdf]

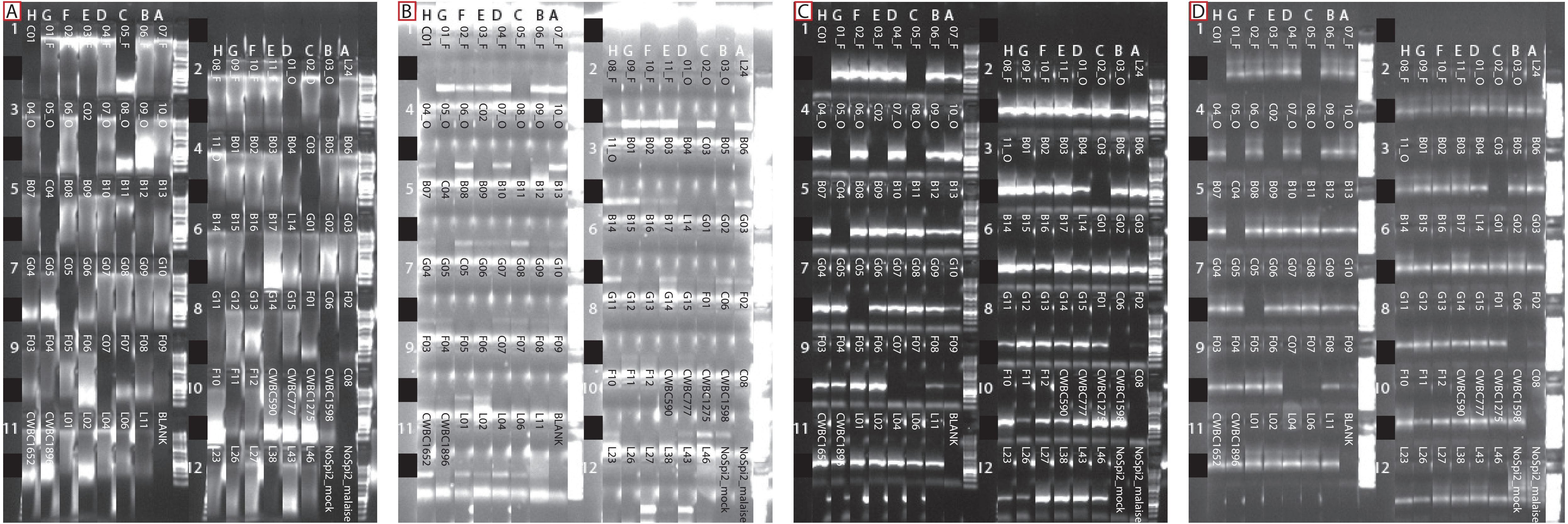

Figure S4: Agarose gel pictures of the 96 well DNA extraction (**A**), PCR1 (**B**), PCR2 (**C**) and normalization (**D**).
